# Imaging dose and secondary cancer risk in image-guided radiotherapy of pediatric patients

**DOI:** 10.1186/s13014-018-1109-8

**Published:** 2018-09-05

**Authors:** Yvonne Dzierma, Katharina Mikulla, Patrick Richter, Katharina Bell, Patrick Melchior, Frank Nuesken, Christian Rübe

**Affiliations:** Department of Radiotherapy and Radiation Oncology, Saarland University Medical Centre, Kirrberger Str. Geb. 6.5, 66421 Homburg, Saar Germany

**Keywords:** Secondary cancer risk, Radiotherapy for Hodgkin lymphoma, Image-guided radiotherapy, Imaging dose in pediatric patients

## Abstract

**Background:**

Daily image-guided radiotherapy (IGRT) can contribute to cover extended body volumes with low radiation dose. The effect of additional imaging dose on secondary cancer development is modelled for a collective of children with Morbus Hodgkin.

**Methods:**

Eleven radiotherapy treatment plans from pediatric patients with Hodgkin’s lymphoma were retrospectively analyzed, including imaging dose from scenarios using different energies (kV/MV) and planar/cone-beam computed tomography (CBCT) techniques. In addition to assessing the effect of imaging dose on organs at risk, the excess average risk (EAR) for developing a secondary carcinoma of the lung or breast was modelled.

**Results:**

Although the variability between the patients is relatively large due to the different target volumes, the additional EAR due to imaging can be consistently determined. For daily 6MV CBCT, the EAR for developing a secondary cancer at age 50 is over 3 cases per 10^4^ PY (patient-years) for the female breast and 0.7–0.8 per 10^4^ PY for the lungs. This can be decreased by using only planar images (< 1 per 10^4^ PY for the breast and 0.1 for the lungs). Similar values are achieved by daily 360° kV CBCT (0.44–0.57 per 10^4^ PY for the breast and 0.08 per 10^4^ PY for the lungs), which is again reduced for daily 200° kV CBCT (0.02 per 10^4^ PY for the lungs and 0.07–0.08 per 10^4^ PY for the breast). These values increase if an older attained age is considered (e.g., for 70 years, by a factor of four for the lungs).

**Conclusions:**

Daily imaging can be performed with an additional secondary cancer risk of less than 1 per 10^4^ PY if kV CBCT is applied. If MV modalities must be chosen, a similar EAR can be achieved with planar images. A further reduction in risk is possible if the imaging geometry allows for sparing of the breast by a partial rotation underneath the patient.

## Background

Modern radiotherapy offers ever improved techniques for optimal target coverage associated with utmost sparing of neighbouring tissues and organs at risk (OAR’s). As dose conformity and dose gradients are increasing, image-guided radiotherapy (IGRT) is a prerequisite, and frequent to daily image-guided positioning verification is common. Since most IGRT systems rely on ionizing radiation for EPID-based (electron portal imaging device) projection or cone-beam computed tomography (CBCT) imaging, an evaluation of the contribution of imaging dose to the treatment plan should be performed, and has been presented for a number of indications in the recent literature. This need is particularly pronounced since there exists a variety of different imaging systems using different photon energy (kV or MV), with 2D or 3D imaging, the dose of which is generally not included in the treatment planning system (TPS). While it is undisputed that the general benefit of image-guided positioning surmounts the possibly deteriorating effects of additional imaging dose on plan quality, particularly sensitive patient collectives such as pediatric patients with good prognosis should not receive excessive imaging dose to avoid OAR complications or – in the even lower dose regime – risk of developing secondary malignancies.

The aim of this study is therefore to analyse and compare several common linac-based imaging scenarios (6MV and 121 kV energies for planar and CBCT imaging with daily or non-daily frequency) with regard to their influence on OAR dose and secondary cancer risk in children with Morbus Hodgkin irradiated at the Saarland University Medical Centre. This collective was chosen because of good long-term survival of children with Hodgkin’s lymphoma, so that there is a high probability that they will live long enough so that a secondary cancer might develop. Furthermore, secondary cancer induction by irradiation of Morbus Hodgkin patients has been extensively studied for the same reason, so that mathematical model parameters for secondary cancer risk are better known for this type of irradiation.

To assess imaging dose contribution, the summation dose from the complete treatment including different imaging scenarios is calculated and secondary cancer risk is estimated. Although other studies have previously estimated secondary cancer risk for the irradiation of children, they have mainly concentrated on the imaging dose or treatment dose separately. This approach is problematic since the secondary cancer risk from these two contributions cannot be expected to be additive – in fact, the secondary cancer risk estimated for imaging dose would normally use a linear model, which only applies in the low-dose regime. Conversely, the secondary cancer risk model for higher doses of the order of a radiotherapy treatment protocol is generally expected to fall out of the linear range, where either bell-shaped or plateau-like models are assumed to account for cell sterilization. In these cases, adding a linearly estimated secondary cancer risk from imaging dose to the risk calculated for the treatment plan separately might yield quite a different result than directly estimating the total risk from the combined dose distribution. A priori, it cannot be known how the two effects interact, which will also be discussed in this study.

## Methods

### Patient collective

We included in the analysis all children treated for Hodgkin’s lymphoma at the Department of Radiotherapy and Radiation Oncology, Saarland University Medical Centre, in the years 2011 through 2015, which are seven children aged 5–17 years (mean age 14 years) with 11 different target sites (analysed separately, see Table 1 for details). After obtaining written informed consent of the patients and the patients' parents, the patients received involved-field irradiation with 19.8–29.8 Gy in fractions of 1.0–1.8 Gy using an intensity modulated radiotherapy technique (IMRT) with 6 MV photons. In total 134 treatment fractions were delivered, with image-guidance in 63 fractions.

**Table 1:** Patients and target volumes analysed. PTV: planning target volume

Case	Patient	Age at treatment	PTV	Prescription	Imaging
1	1	16	1st series: right lung; 2nd series: right lung and infraclavicular/pectoral/ mediastinal lymphatics	1st series: 3x100cGy; 2nd series: 11 × 100 cGy; 11x166cGy	1 x 6MV axes 11 x IBL axes
2	2	15	bilateral neck cervical, supra- and infraclavicular lymphatics and mediastinum	11x180cGy	1 x 6MV axes 3 x IBL axes
3	3	17	Right cervical/mediastinal lymphatics	11x180cGy	4 x 6MV axes 1 x 6MV CBCT (7.2 MU)
4	4	16	1st series: Mediastinum 2nd series: Boost Mediastinum	1st series: 11x180cGy; 2nd series: 5x200cGy	1 x 6MV axes 4 x IBL axes
5	4	16	Spleen	11x180cGy	1 x 6MV axes 4 x IBL axes
6	5	16	1st series: Mediastinum; 2nd series: Boost macroscopic residual disease (Mediastinum)	1st series: 11x180cGy; 2nd series: 5x200cGy	6 x kV axes 1 x kV CBCT (414 mAs)
7	5	16	Spleen	11x180cGy	1 x 6MV/IBL axes 1 x kV axes 1 x kV CBCT (621 mAs)
8	6	5	bilateral neck, supra-infraclavicular, mediastinal and paraaortic lymphatics, spleen	11x180cGy	3 x 6MV axes 5 x 6MV CBCT (54 MU in total)
9	6	5	Os ileum	11x180cGy	5 x 6MV portal images
10	7	13	Left cervical lymphatics	11x180cGy	1 x kV axes 2 x kV CBCT (458 mAs in total)
11	7	13	Spleen	11x180cGy	2 x kV axes 3 x kV CBCT (1103 mAs in total) 1 x IBL CBCT (11 MU)

For image-guidance, the following systems are available at our department:

1. 6 MV treatment beam (TBL) at two Siemens Artiste and one Siemens Oncor (Siemens Healthcare, Erlangen, Germany) linear accelerators,
2. 1 MV image beam line (IBL) at two Siemens Artiste linear accelerators [1–3], and
3. 121 kV system kVision at one Siemens Artiste linear accelerator [4–7].

All systems can be used to acquire planar or CBCT images. Planar MV axial images are taken using 1 monitor unit (MU) each from two orthogonal views. For MV CBCT images, either a full 360° or a shortened arc (200°) can be used with different MU settings depending on the patient anatomy and geometry (7–11 MU), with a square field of 27.4 cm width at source-to-surface distance (SSD) 100 cm [1]. The kVision system applies a “pre-shot” to automatically optimize the exposure (the mAs value is displayed and protocolled, generally less than 10 mAs for planar images and between 200 and 700 mAs for CBCT in our collective). While the MV CBCT gantry rotates above the head of the patient, the kV CBCT geometry with a reduced arc is inverted, rotating below the back of the patient because the X-ray tube is installed opposite the treatment head. The field size at SSD 100 cm is 28 × 28 cm^2^ for the kV system [4].

**Fig. 1:**
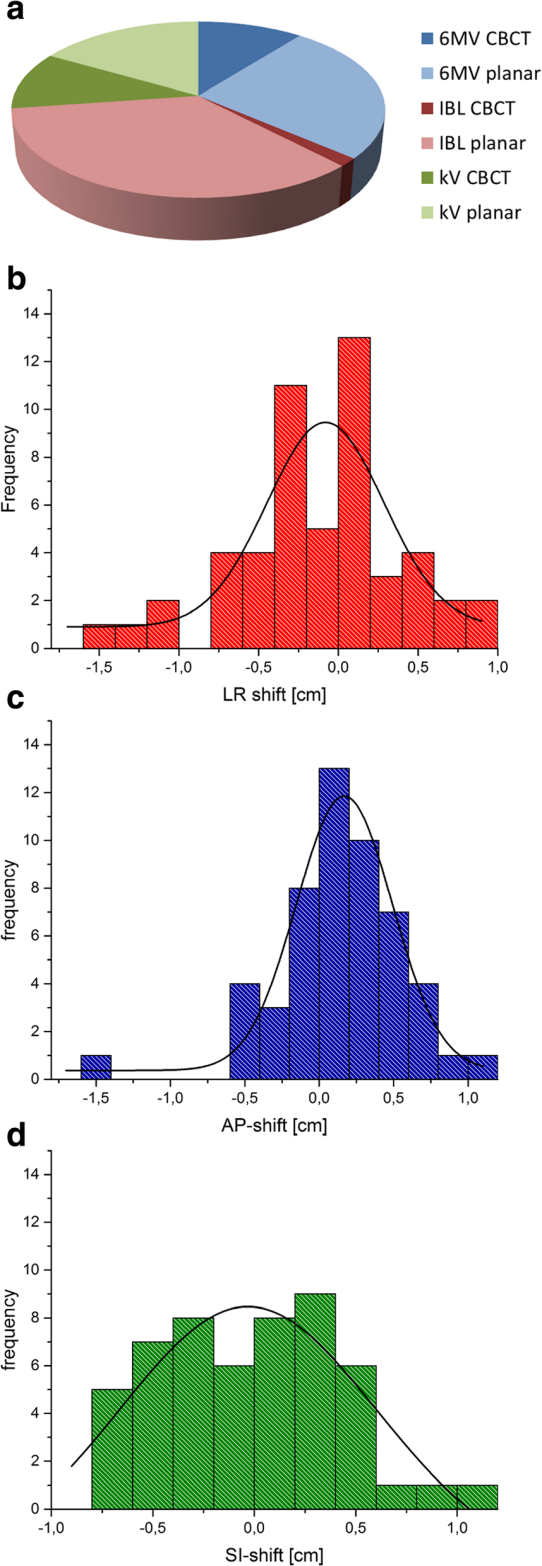
**a** Percentage use of each imaging modality in the patient collective. **b** Frequency distribution of couch shifts in left-right (LR), (**c**) anterior-posterior (AP) and (**d**) superior-inferior (SI) direction

The realistic imaging scenarios applied for the patients depended to some degree on the availability of the techniques for imaging – in 2011, only the 6 MV energy was available, the kVision system was installed last (end 2012). When available, the lower-energy systems were preferentially used for imaging; the percentage use of each imaging system is shown in Fig. 1a. A no-action-level protocol of online positioning correction was followed, so that all deviations observed in pre-treatment imaging (unless smaller than 1 mm) were always corrected for. An analysis of the performed couch shifts after imaging (Fig. 1b) agrees with a normal distribution, although the patient collective is too small to allow for statistical significance. It was checked in a phantom study that the different imaging modalities are in agreement regarding the detected positioning errors [8].

### Dose calculation and secondary cancer risk model

The three imaging systems are all commissioned in the Philips Pinnacle treatment planning system (Philips Healthcare, Koninklijke, Netherlands) [1, 4, 9], so that the imaging dose can be calculated together with the patient treatment plan. For each patient, the dose distribution of the original treatment plan (as it was accepted for treatment = Scenario 1) without inclusion of imaging dose is compared with the following imaging scenarios:

- Scenario 2: treatment plan with real imaging performed for the patient, with imaging device and frequency differing between the individual patients (details listed in Table 1)
- Scenario 3: treatment plan with daily 121 kV 200° CBCT
- Scenario 4: treatment plan with daily 121 kV 360° CBCT
- Scenario 5: treatment plan with daily 6 MV planar imaging
- Scenario 6: treatment plan with daily 360° 6 MV CBCT.

The hypothetical scenarios 3–6 are chosen to analyse the effect of daily imaging, which is more and more becoming the norm [10]. For the kV and 6 MV CBCT scenarios, the average mAs or MU per rotation was calculated for all patients and applied for the hypothetical scenarios: 360° kV CBCT with 377 mAs, 200° kV CBCT with 99 mAs, 6 MV CBCT (360°) with 11 MU. Evidently, kV imaging is the most desirable scenario; however, this is not available at every clinic. This is why we include 6 MV imaging to see how daily imaging can be achieved if only this modality is available. While it is clear that daily 6 MV CBCT would entail too high imaging dose, we include this scenario as the maximum possible imaging dose; contrarily, daily planar images with 6 MV are included as example of the lowest achievable imaging dose for daily MV images. Reality will range in between these two scenarios as occasional CBCT may be used to acquire 3D views. As kV planar images involve very little dose in comparison with all other modalities (lower than kV CBCT or planar MV images by at least an order of magnitude), this modality is not included in our analysis.

Dose calculation of the summation plans was carried out in the Philips Pinnacle TPS Version 9.0–9.6 on a 2 mm dose grid using the collapsed cone algorithm (for a detailed explanation of the procedure, compare [11]). Dose-volume histogram (DVH) values are considered to assess dose to the organs at risk (OARs). For secondary cancer risk calculation, the dose distribution was exported together with the regions of interests (ROIs) of the OARs and imported into Matlab R2015a (MathWorks, Natick, Massachusetts, USA). An in-house software was created to calculate the organ-specific risk for developing secondary carcinoma using Schneider’s mechanistic model [12]. This gives the organ excess absolute risk as

$$ {EAR}_{org}\left({age}_x,{age}_a\right)={\beta}_{\mathrm{o} rg}\bullet OED\bullet \mu \left({age}_x,{age}_a\right), $$

where *age*_*x*_ and *age*_*a*_ are the age at exposure and age attained (when developing the secondary malignancy), respectively,

$$ \mu \left({age}_x,{age}_a\right)=\exp \left({\gamma}_e\left({age}_x-30\right)+{\gamma}_a\ln \frac{age_a}{70}\right) $$

is a modifying function for adjusting the risk to the ages *age*_*x*_ and *age*_*a*_ and *β* is a model scaling parameter between the organ effective dose (OED) and the EAR. The organ effective dose is calculated according to [12] as the risk-equivalent-dose (RED)-weighted average of the total volume *V*_*T*_

$$ OED=\frac{1}{V_T}\bullet \sum \limits_iV\left({D}_i\right) RED\left({D}_i\right), $$

with the organ-specific dose-response relationship given by the mechanistic model for carcinoma induction including cell killing and fractionation effects:

$$ RED(D)=\frac{\exp \left(-{\alpha}^{\prime }D\right)}{\alpha^{\prime }R}\left(1-2R+{R}^2\exp \left({\alpha}^{\prime }D\right)-\left(1-{R}^2\right)\exp \left(-\frac{\alpha^{\prime }R}{1-R}\right)D\right). $$

Fractionation is taken into account using the linear quadratic model with parameters *α* and *β* and a fractionation with target volume prescribed dose *D*_*T*_ in fraction doses of *d*_*T*_:

$$ {\alpha}^{\prime }=\alpha +\beta d=\alpha +\beta \frac{D}{D_T}{d}_T. $$

The main two organs at risk for secondary cancer development in our patient collective are the lungs and the breast, for which the model parameters are given in Table 2.

**Table 2:** Parameters for modelling the excess absolute risk (from [12])

Model parameter	Female breast	Lung
*α* in Gy^−1^	0.044	0.042
R	0.15	0.83
*β*_*org*_ in excess cases per 10,000 PY Gy	8.2	8.0
*γ*_*e*_	−0.037	0.002
*γ*_*a*_	1.7	4.23

The software uses the DICOM (Digital Imaging and Communications in Medicine) structure sets, RT dose and CT data set as input together with the organ specific model parameters to calculate the excess absolute cancer risk. In each case, the age of the patient at exposure was included; the secondary cancer risk was modelled for an attained age of 50 years.

A statistical analysis was carried out in Origin Pro 2015G (OriginLab, Northampton, Massachusetts, USA) for descriptive statistics. To assess differences between the planning scenarios, the plans were pair-wise compared against the original plan (“gold standard” without imaging dose) using the Wilcoxon signed-rank test. Please note that “gold standard” is taken to mean the optimal dose distribution (no deterioration from imaging dose), as it was accepted for treatment – this is *not* suggested as the optimal verification scenario, just as a baseline for dose comparisons (see Discussion).

## Results

### Visualization of dose distributions – Example

**Fig. 2:**
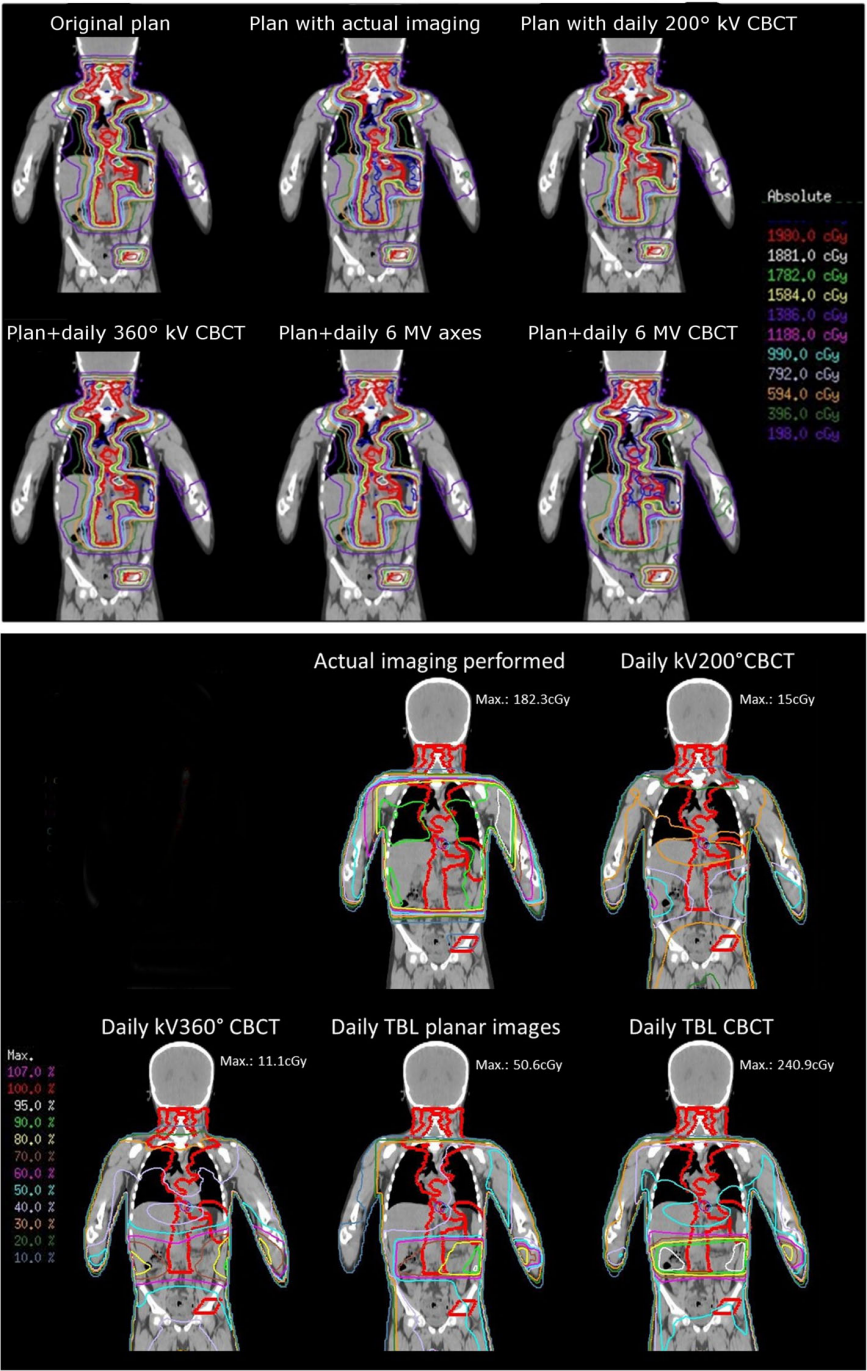
Upper panel: dose distribution for the original treatment plan and summation plan including different imaging scenarios. Lower panel: dose distribution for different imaging scenarios only

Figure 2 shows an example of the imaging dose distribution and the planned dose, where differences between the imaging scenarios become apparent. We selected this patient example because several issues can be observed here: Firstly, the dose distribution from imaging only differs strongly from the original treatment plan. Secondly, the dose distributions of the different imaging scenarios are quite different from each other, both in absolute dose (all imaging dose distributions are scaled to their respective maximum dose, ranging from 11.1 cGy for daily kV CBCT to 240.9 cGy in total for daily 6 MV CBCT) and in dose distribution. For this patient (patient 6), two target volumes were irradiated (lymphatics and os ileum), so that two series of imaging scenarios were simulated. The overlapping region between both imaging sets creates a higher-dose “belt” in the simulated scenarios. In the actual verification, most sessions only imaged the larger cranial planning target volume (PTV), so that most imaging dose is accumulated here. Furthermore, the actual verification used a combination of axial and CBCT imaging, which explains the square shape of the isodoses in this scenario.

If the combined dose from the radiotherapy treatment and imaging is considered, the differences between the scenarios are less obvious. Some small increases in the lower-dose isolines and an increased maximum dose are observed, particularly for those imaging scenarios with higher additional dose (e.g., 6 MV). For this patient, the actual imaging performed contributed rather high dose because this patient was treated among the first in the collective, when kV imaging was not yet installed at our department.

### Dose to organs at risk – DVH analysis

**Fig. 3:**
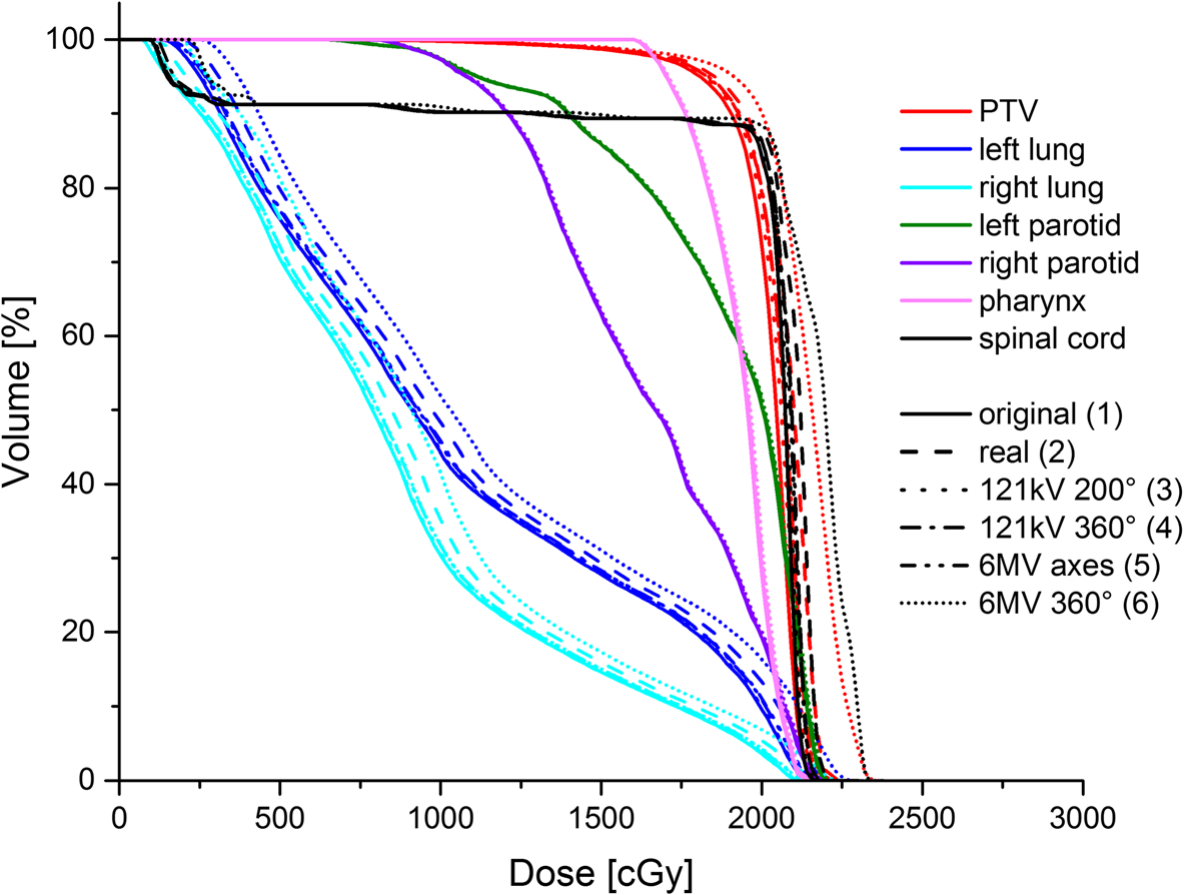
Example DVH for original plan and different imaging scenarios (same patient as in Fig. 2)

An example DVH for the patient shown above is given in Fig. 3. It is obvious that the dose is markedly increased by daily 360° 6MV CBCT, as well as (to a smaller degree) by the actual mixed CBCT-planar 6 MV imaging performed. The other imaging scenarios do not result in a visible increase in the DVH dose. The fact that neither dose to the parotids nor the pharynx appear to change with the imaging scenarios is due to the imaging geometry visible in Fig. 2, in which the imaging beam does not reach up as far cranially to involve these organs.

**Fig. 4:**
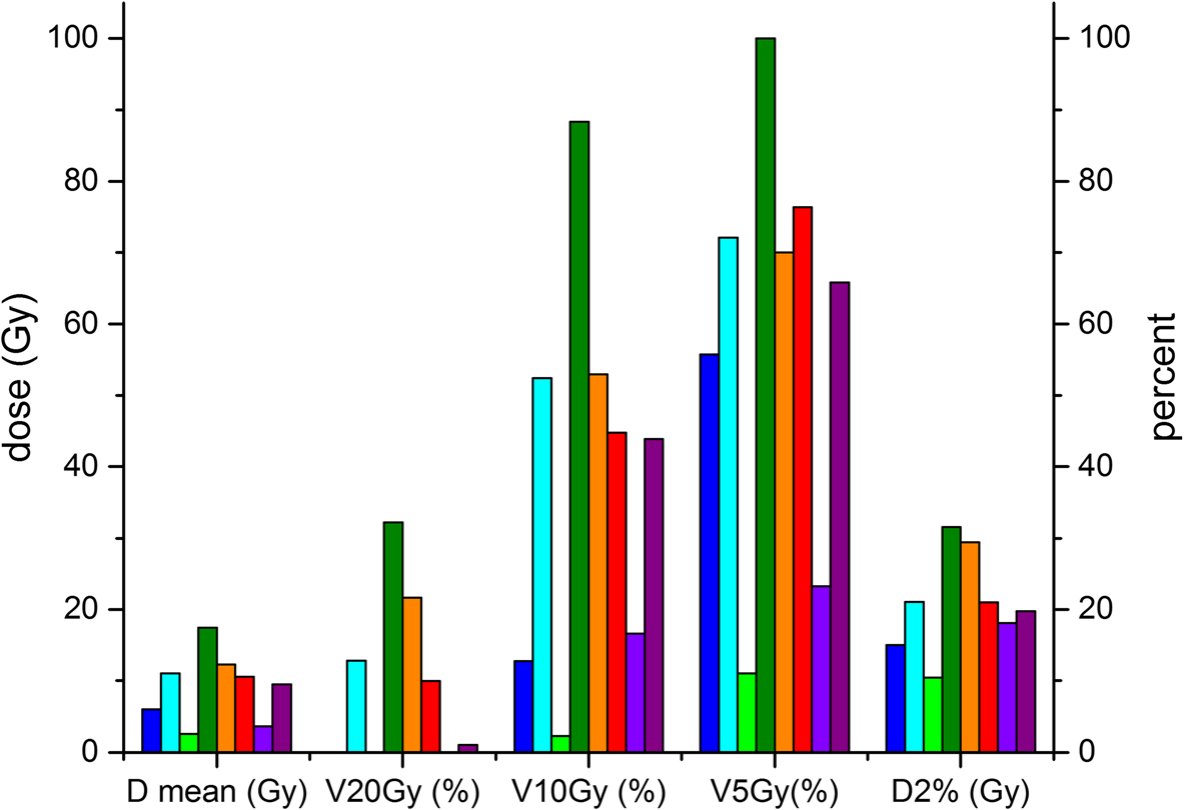
Dose metrics for the left lung (all patients). Shown are 8 separate data sets because one patient received two separate planning CTs for the two target sites

All DVH parameters are listed in Table 3; an example for the left lung is displayed in Fig. 4. The statistical tests for significance using Wilcoxon’s signed-rank test for paired data gave positive results for all organs for which more than 5 patient cases with DVH values existed, because the comparison of any plan with imaging vs. the original plan without imaging always gave positive ranks. Only for those DVH measures such as the D20Gy for the parotid could significance not be reached because of the variability of the plans, which meant that more than half of the patients had D20Gy = 0% for this organ.

**Table 3:** Dose metrics for the organs at risk in the imaging scenarios (mean ± standard deviation, range)

Organ	Criteria	Scenario1	Scenario 2	Scenario 3	Scenario 4	Scenario 5	Scenario 6
Lung le	D mean [Gy]	9.13 ± 4.88 (2.62–17.43)	9.27 ± 4.90 (2.74–17.54)	9.17 ± 4.90 (2.65–17.53)	9.28 ± 4.96 (2.74–17.80)	9.39 ± 5.01 (2.81–17.98)	10.46 ± 5.62 (3.59–20.25)
	V20Gy [%]	9.72 ± 12.07 (0.00–32.26)	11.04 ± 12.55 (0.00–32.55)	9.91 ± 12.20 (0.00–32.54)	10.33 ± 12.52 (0.00–33.34)	10.73 ± 12.74 (0.00–33.84)	14.63 ± 15.05 (0.00–40.55)
	V10Gy [%]	39.27 ± 27.78 (2.33–88.37)	40.03 ± 27.99 (2.44–88.88)	39.46 ± 27.92 (2.35–88.85)	39.89 ± 28.27 (2.43–90.16)	40.36 ± 28.54 (2.49–91.08)	44.55 ± 30.80 (3.20–98.92)
	V5Gy [%]	59.29 ± 29.06 (11.03–100)	60.2 ± 29.24 (11.92–100)	59.46 ± 29.06 (11.23–100)	59.9 ± 28.97 (11.92–100)	60.38 ± 29.01 (12.38–100)	67.28 ± 30.31 (20.45–100)
	D 2% [Gy]	20.81 ± 6.98 (3.33–31.78)	20.96 ± 6.96 (3.43–31.84)	20.85 ± 6.99 (3.37–31.84)	20.98 ± 7.03 (3.45–32.05)	21.14 ± 7.07 (3.47–32.11)	22.12 ± 7.39 (4.33–34.14)
Lung ri	D mean [Gy]	9.2 ± 6.10 (1.58–18.66)	9.33 ± 6.12 (1.65–18.72)	9.24 ± 6.12 (1.61–18.74)	9.29 ± 6.08 (1.68–18.60)	9.38 ± 6.18 (1.65–18.99)	10.43 ± 6.62 (2.26–20.98)
	V20Gy [%]	10.1 ± 13.08 (0.00–38.70)	10.6 ± 13.14 (0.00–38.89)	10.2 ± 13.68 (0.00–38.92)	10.62 ± 13.44 (0.00–39.56)	10.9 ± 13.52 (0.00–39.67)	15.32 ± 16.26 (0.00–46.67)
	V10Gy [%]	41.13 ± 35.44 (0.00–98.93)	41.86 ± 35.30 (0.00–98.94)	41.26 ± 35.47 (0.00–98.93)	41.52 ± 35.55 (0.00–98.95)	41.70 ± 35.51 (0.00–98.96)	45.4 ± 36.36 (0.00–99.12)
	V5Gy [%]	56.29 ± 35.96 (0.00–100)	57.13 ± 36.30 (0.00–100)	56.45 ± 35.96 (0.00–100)	56.8 ± 35.90 (0.00–100)	57.03 ± 35.95 (0.00–100)	62.4 ± 36.23 (0.00–100)
	D 2% [Gy]	20.5 ± 8.66 (3.33–31.78)	20.64 ± 8.65 (3.43–31.84)	20.54 ± 8.67 (3.37–31.48)	20.65 ± 8.70 (3.45–32.05)	20.72 ± 8.73 (3.47–32.11)	21.72 ± 9.24 (4.33–34.14)
Breast le	D mean [Gy]	3.10 ± 2.84 (0.74–7.06)	3.19 ± 2.81 (0.87–7.10)	3.13 ± 2.86 (0.76–7.11)	3.29 ± 2.95 (0.89–7.44)	3.38 ± 3.01 (0.94–7.62)	4.27 ± 3.53 (1.60–9.34)
	D 2% [Gy]	12.60 ± 5.65 (8.85–20.89)	12.67 ± 5.53 (9.01–20.75)	12.56 ± 5.59 (8.86–20.75)	12.76 ± 5.66 (9.02–21.05)	12.86 ± 5.70 (9.08–21.21)	13.93 ± 6.12 (9.90–22.89)
Breast ri	D mean [Gy]	5.81 ± 4.80 (1.37–11.53)	5.89 ± 4.82 (1.41–11.67)	5.83 ± 4.81 (1.38–11.56)	6.0 ± 4.85 (1.49–11.73)	6.01 ± 4.86 (1.50–11.75)	6.97 ± 5.13 (2.16–12.82)
	D 2% [Gy]	16.08 ± 6.12 (9.84–21.41)	16.18 ± 6.12 (9.90–21.43)	16.09 ± 6.12 (9.85–21.43)	16.28 ± 6.17 (9.99–21.43)	16.34 ± 6.19 (10.03–21.70)	17.41 ± 6.46 (10.83–23.31)
Parotid le	D mean [Gy]	5.25 ± 7.94 (0.17–18.71)	5.29 ± 7.92 (0.20–18.75)	5.28 ± 7.93 (0.20–18.71)	5.36 ± 7.91 (0.27–18.72)	5.38 ± 7.92 (0.21–18.72)	5.98 ± 7.82 (0.55–18.78)
	D 2% [Gy]	6.68 ± 9.76 (0.24–21.78)	6.74 ± 9.53 (0.36–21.82)	6.71 ± 9.75 (0.28–21.78)	6.66 ± 9.70 (0.40–21.78)	6.86 ± 9.71 (0.41–21.79)	7.62 ± 9.52 (1.20–21.86)
Parotid ri	D mean [Gy]	3.77 ± 5.96 (0.39–16.50)	3.81 ± 5.95 (0.43–16.53)	3.80 ± 5.95 (0.42–16.50)	3.89 ± 5.92 (0.50–16.51)	3.90 ± 5.92 (0.45–16.51)	4.51 ± 5.74 (0.77–16.57)
	D 2% [Gy]	5.50 ± 7.92 (0.62–21.41)	5.48 ± 7.92 (0.73–21.46)	5.48 ± 7.91 (0.66–21.42)	5.58 ± 7.88 (0.78–21.43)	5.59 ± 7.87 (0.79–21.43)	6.37 ± 7.60 (1.59–21.49)
	OED	0.79 ± 0.82 (0.14–2.20)	0.81 ± 0.81 (0.20–2.20)	0.81 ± 0.82 (0.16–2.20)	0.84 ± 0.80 (0.22–2.20)	0.84 ± 0.80 (0.22–2.20)	1.05 ± 0.70 (0.36–2.20)
	EAR	0.43 ± 0.47 (0.08–1.31)	0.43 ± 0.47 (0.09–1.31)	0.43 ± 0.47 (0.09–1.28)	0.45 ± 0.46 (0.11–1.31)	0.45 ± 0.46 (0.10–1.31)	0.55 ± 0.42 (0.16–1.31)
Pharynx	D mean [Gy]	6.51 ± 7.52 (0.72–19.34)	6.56 ± 7.50 (0.84–19.38)	6.53 ± 7.49 (0.75–19.34)	6.62 ± 7.49 (0.87–19.35)	6.66 ± 7.48 (0.93–19.36)	7.38 ± 7.28 (2.05–19.43)
	D 2% [Gy]	12.28 ± 7.29 (1.06–21.05)	12.37 ± 7.24 (1.18–21.11)	12.31 ± 7.28 (1.09–21.06)	12.48 ± 7.25 (1.22–21.06)	12.39 ± 7.19 (1.27–21.07)	13.33 ± 6.95 (2.41–21.15)
Spinal cord	D 2% [Gy]	19.66 ± 7.97 (6.36–29.53)	19.8 ± 7.97 (6.46–29.60)	19.71 ± 7.99 (6.40–29.61)	19.81 ± 8.02 (6.48–29.80)	19.89 ± 8.05 (6.50–29.90)	21.17 ± 8.43 (7.41–31.67)

**Fig. 5:**
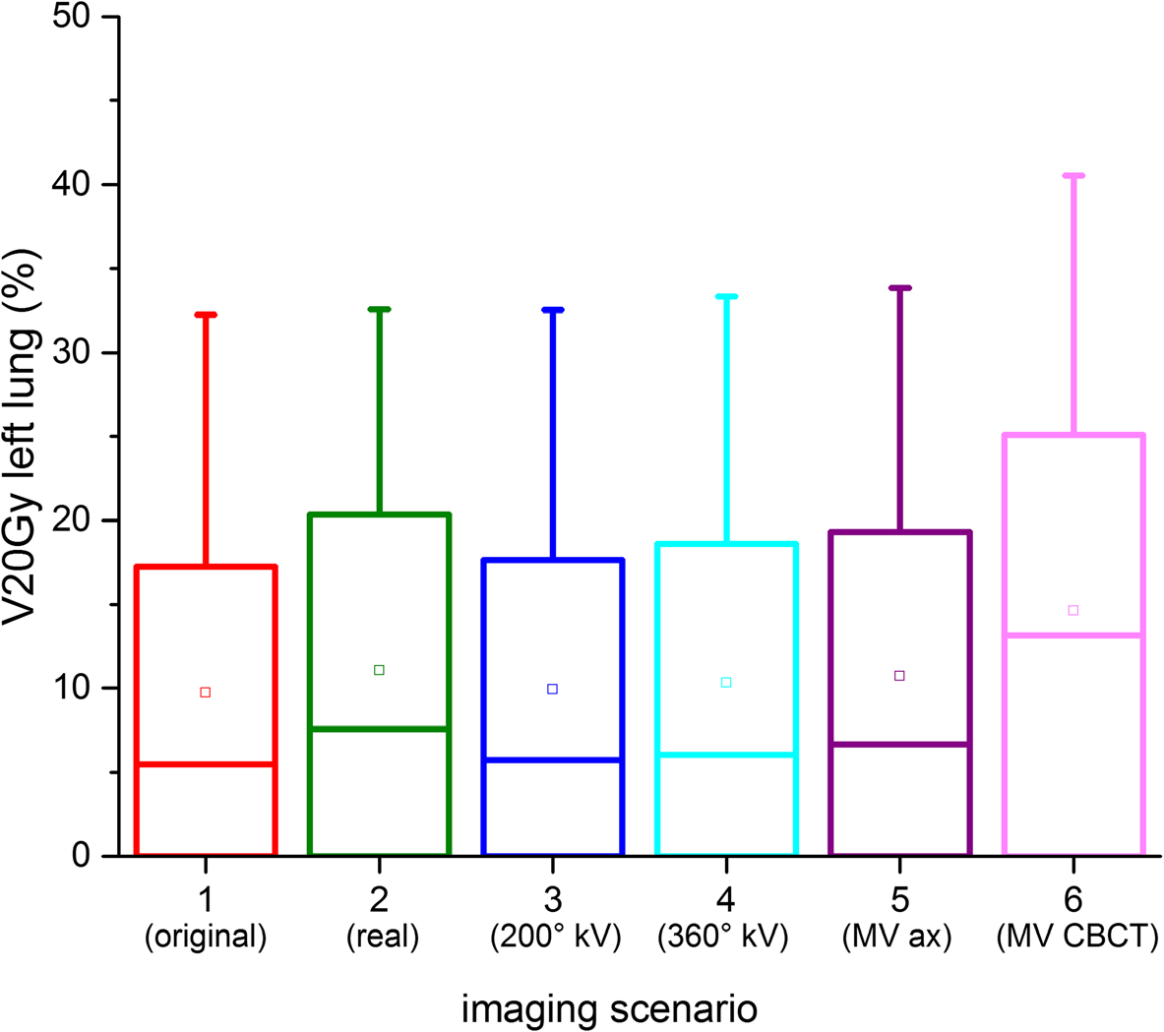
Spread of the V20Gy dose metric for the left lung across the patient collective. The box gives the 25% and 75% quartile, the horizontal line the median, the square the average. Whiskers reach to the maximum

**Fig. 6:**
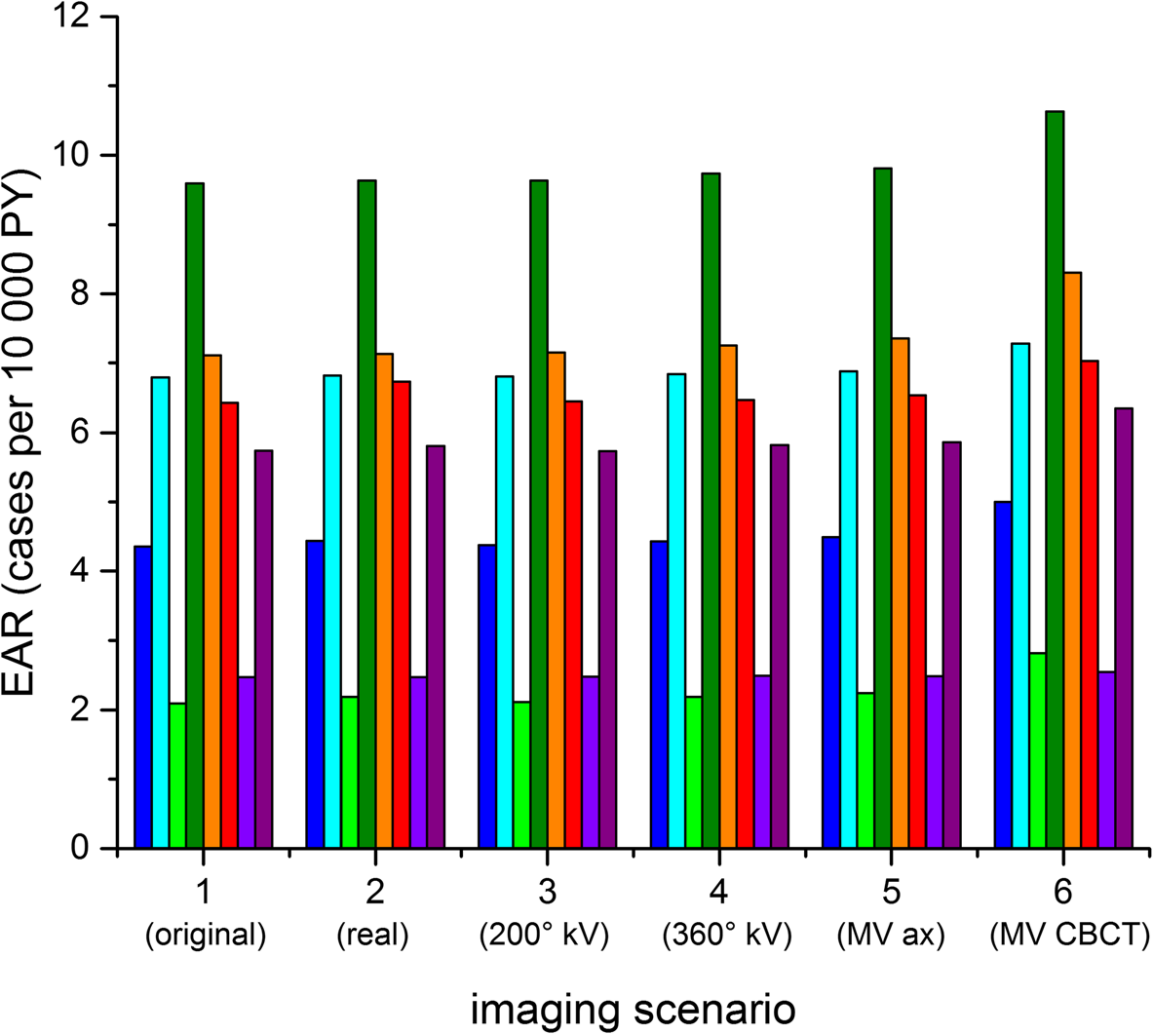
Excess absolute cancer risk for the left lung for the patient collective modelled for different imaging scenarios (each colour bar corresponds to an individual patient CT data set)

As the target volumes and hence treatment plans vary considerably between the patients, the DVH parameters are also very different. Figure 4 shows the variation in DVH parameters for the left lung for the whole patient collective only for the original treatment plan without imaging. The additional dose from imaging creates less absolute difference than the variation between the individual patients, as can be seen for the example of the left lung values in Figs. 5 and 6. For the parotid mean and D2% values, even the extreme imaging scenario with daily 6 MV CBCT only increases dose by less than 1 Gy. For the spinal cord, the D2% dose increases from 19.7 Gy without imaging to at most 21.2 Gy (daily 6 MV), and by only 0.1 Gy for scenarios with kV imaging. Similar results are obtained for the pharynx and maximum (D2%) and mean lung doses. For the lower-dose DVH values, the differences increase somewhat, e.g. by more than 5% for the lung V20Gy and V5Gy. If the individual patient values (range) are  considered rather than the average over the patient collective, strong variations appear, with V20% for the lung ranging from 0% to over 46% and V5Gy from 56 to 100% within the collective.

### Imaging effect on secondary cancer risk

Secondary cancer risk reflects the behavior of DVH values in varying more between individual patients than between imaging scenarios (Fig. 6, Table 4). Considering the original plan only (no imaging), the absolute excess cancer risk from treatment for the lungs is of the order of 5–6 cases per 10^4^ PY (range 1.3–10 per 10^4^ PY) and for the breast ranges between 2.4 and 26 per 10^4^ PY.

**Fig. 7:**
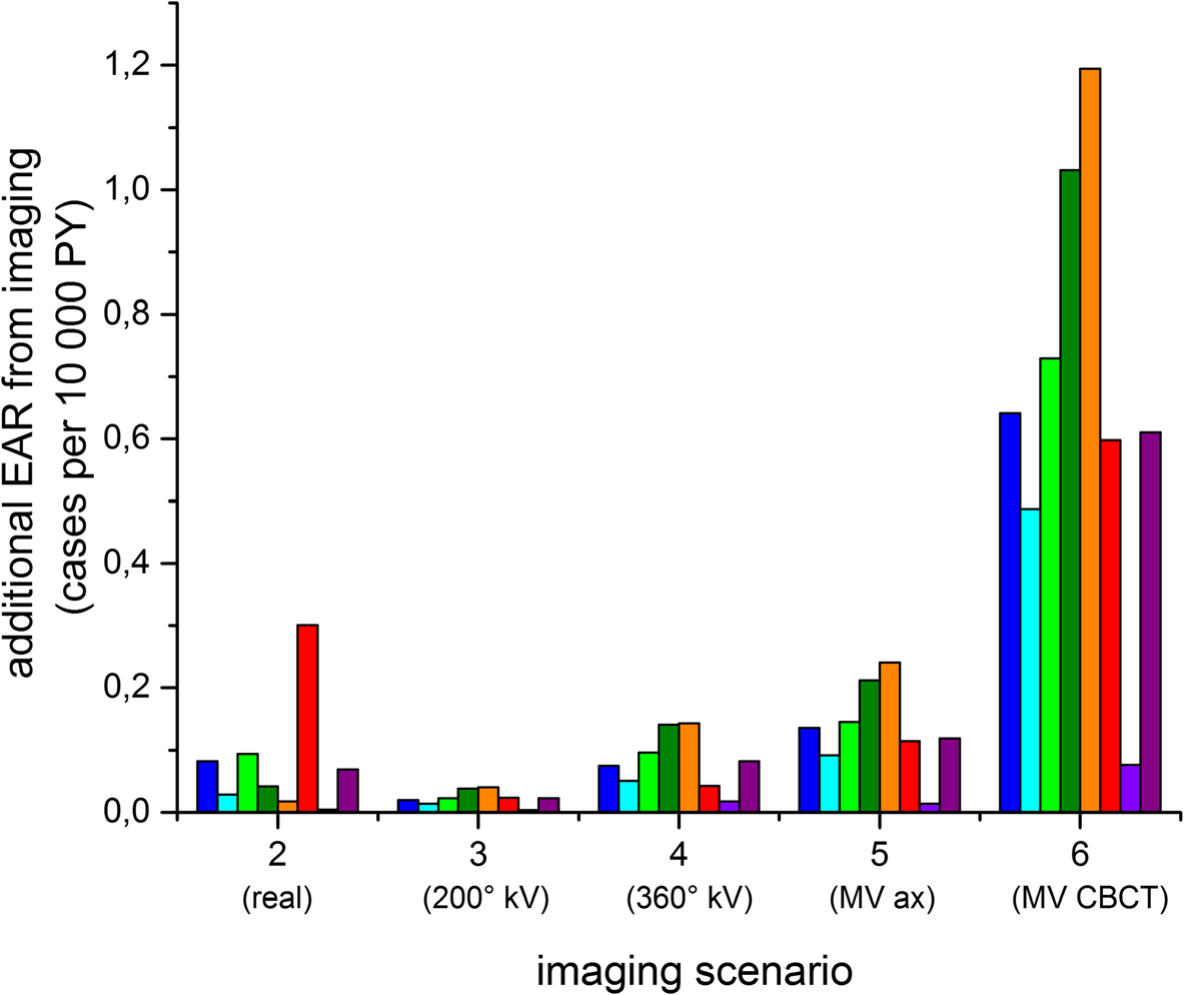
Additional EAR from imaging only (difference between total EAR as shown in Fig. 6 and EAR from the original plan, each colour bar corresponds to an individual patient CT data set)

**Table 4:** Excess absolute risk (in cases per 10^4^ PY): average ± standard deviation (min-max)

	Scenario 1 (original)	Scenario 2 (real)	Scenario 3 (200° kV-CBCT)	Scenario 4 (360° kV-CBCT)	Scenario 5 (6MV axes)	Scenario 6 (6MV CBCT)
Left lung	5.58 ± 2.5 (2.1–9.6)	5.66 ± 2,5 (2.2–9.6)	6.25 ± 2.7 (2.6–10.6)	5.72 ± 2.6 (2.2–9.9)	5.65 ± 2.5 (2.2–9.7)	5.60 ± 2.5 (2.1–9.6)
Right lung	5.54 ± 3.2 (1.3–10.0)	5.62 ± 3,2 (1.4–10.0)	6.17 ± 3.3 (1.9–10.8)	5.64 ± 3.2 (1.4–10.1)	5.62 ± 3.2 (1.4–10.1)	5.56 ± 3.2 (1.3–10.0)
Left breast	8.73 ± 6.9 (2.4–17.9)	9.03 ± 6,8 (2.8–18.0)	11.87 ± 7.7 (5.3–22.7)	9.53 ± 7.1 (3.0–19.2)	9.30 ± 7.0 (2.9–18.8)	8.81 ± 6.9 (2.4–18.0)
Right breast	14.12 ± 10.3 (4.8–26.0)	14.29 ± 10.2 (5.0–26.2)	16.63 ± 10.0 (7.7–27.5)	14.56 ± 10.2 (5.3–26.3)	14.57 ± 10.2 (5.3–26.3)	14.19 ± 10.2 (4.9–26.1)

**Fig. 8:**
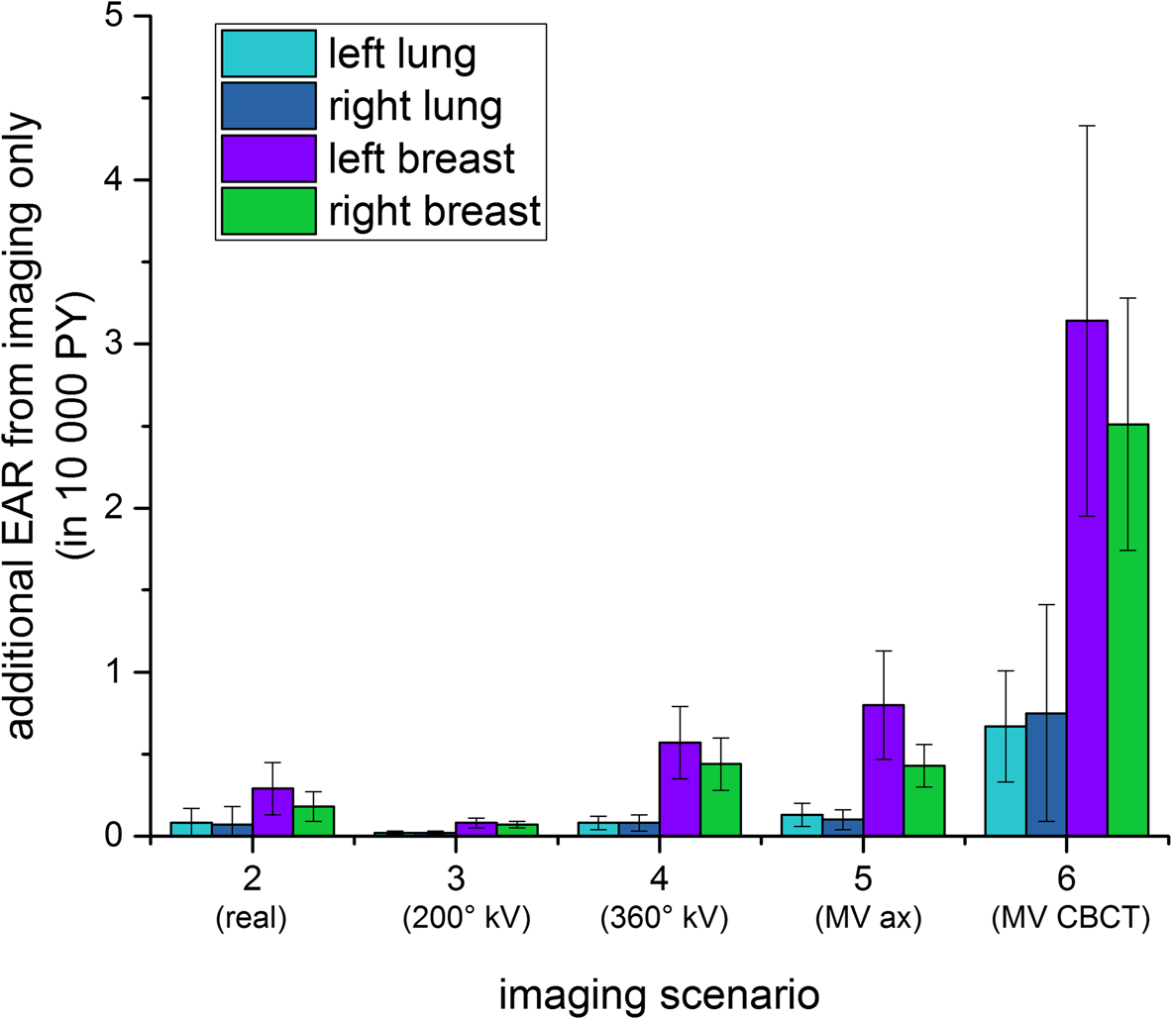
Additional EAR from imaging only, average (with standard deviation) over all patients

To concentrate on the effect of imaging rather than the inter-individual differences in target volume, we consider the differences between the original plan and the plans including imaging. The absolute excess risk from imaging only (disregarding the “baseline risk” from radiotherapy treatment) is given in Table 5 and shown in Fig. 7 for the left lung. Comparing this with Fig. 6 shows that the variability between the patients is drastically decreased, so that the systematic effect from imaging becomes visible. We can therefore now calculate the average additional risk over the patient collective (Fig. 8), which is considered in the following.

**Table 5:** Additional EAR from imaging only (in cases per 10^4^ PY, average ± standard deviation, range in braces)

Organ	Scenario 2 (real)	Scenario 3 (200° kV-CBCT)	Scenario 4 (360° kV-CBCT)	Scenario 5 (6MV axes)	Scenario 6 (6MV CBCT)
Left lung	0.08 ± 0.09 (0.00–0.30)	0.02 ± 0.01 (0.00–0.04)	0.08 ± 0.04 (0.02–0.14)	0.13 ± 0.07 (0.01–0.24)	0.67 ± 0.34 (0.08–1.19)
Right lung	0.07 ± 0.11 (0.00–0.07)	0.02 ± 0.01 (0.00–0.04)	0.08 ± 0.05 (0.02–0.16)	0.1 ± 0.06 (0.01–0.21)	0.75 ± 0.66 (0.08–2.31)
Left breast	0.29 ± 0.16 (0.11–0.46)	0.08 ± 0.03 (0.06–0.12)	0.57 ± 0.22 (0.38–0.88)	0.8 ± 0.33 (0.55–1.29)	3.14 ± 1.19 (1.99–4.81)
Right breast	0.18 ± 0.09 (0.09–0.31)	0.07 ± 0.02 (0.04–0.09)	0.44 ± 0.16 (0.25–0.64)	0.43 ± 0.13 (0.27–0.58)	2.51 ± 0.77 (1.50–3.33)

As was expected, daily 6 MV CBCT causes most additional cancer risk (more than 3 cases per 10^4^ PY for the female breast and 0.7–0.8 cases per 10^4^ PY for the lungs). If only 6 MV imaging is available, the secondary cancer risk can be drastically decreased by preferentially performing planar images; in the case of daily planar 6 MV imaging the additional excess absolute cancer risk for the breast is decreased to 0.4–0.8 cases per 10^4^ PY (a factor of 3.9–5.8) and to 0.1 cases for the lungs (more than a factor of 5). Slightly lower values are achieved by daily 360° kV CBCT (0.44–0.57 per 10^4^ PY for the breast and 0.08 per 10^4^ PY for the lungs), which is again reduced for daily 200° kV CBCT. For this scenario, the additional excess absolute cancer risk for the lungs is 0.02 per 10^4^ PY (lower than 360° by a factor of 4) and 0.07–0.08 per 10^4^ PY for the breast (decreased by a factor of 6–7 with respect to the 360° kV CBCT scenario). The reason for this more pronounced improvement in the secondary cancer risk for the breast as compared with the lung is the geometry of the imaging system: The 200° kV CBCT is taken rotating the X-ray source under the back of the patient, so that the breast only receives the much attenuated exit dose. As the lungs are located more centrally, the difference between a full CBCT rotation and a partial one does not produce such a pronounced effect. On average, the real imaging performed contributed about as much to secondary cancer risk as daily kV CBCT with mixed 200° and 360° kV CBCT would have.

## Discussion

We have mathematically estimated the risk for developing secondary cancers of the lung and female breast for a collective of eight pediatric patients (11 radiotherapy treatment plans) with Morbus Hodgkin based on the dose distribution from the treatment plan and several clinically relevant IGRT scenarios. Although there is relatively large variability between the patients, the additional risk from imaging could be determined. Depending on the imaging scenario used, the excess average risk at age 50 for developing a secondary cancer of the lungs ranged between 1.3 and 10 cases per 10^4^ PY (0–2.3 per 10^4^ PY only from imaging) and 2.4–27.5 cases per 10^4^ PY (0.1–4.8 per 10^4^ PY from imaging) for a secondary cancer of the female breast.

### Influence of assumed age at secondary cancer incidence

Our estimates for the EAR of secondary cancer development depend on the age attained included in the model. We have opted for a relatively young age of 50 years, since this is already more than 30 years after treatment (age of exposure was set to the real age of the patients at treatment) and since this would be an age at which all possible therapeutic measures would most probably still be taken to cure the secondary malignancy.

The value assumed for the age attained only influences the risk estimate via the modifying function *μ*, which directly scales with the EAR. For our patient collective and an assumed age attained of 50 years, *μ* for the lungs is of the order of 0.23, but strongly increases with age attained to 0.95–0.97 for 70 years and about 2.8 for 90 years. This explains to some degree the relatively small EAR values obtained in our study. By considering an attained age of 70 rather than 50 years, all presented risk values would be increased about fourfold, and up to a factor 12 if a long life expectancy as 90 years is assumed. For breast cancer, *μ* varies more between the patients, as the dependence on the age at exposure is stronger. For an attained age of 50 years, *μ* is between 0.95 and 1.32, increasing to 1.7–2.3 for an attained age of 70 years and to 2.5–3.6 for 90 years. Here, the age attained only increases the estimated EAR by a factor of less than 3.

### Influence of modelling approach

All models for secondary cancer induction are simplified estimates based on the available evidence, which is complicated by the long latency periods and the existence of a large number of confounding factors influencing the risk of secondary cancer induction (e.g., additional chemotherapy, life-style and other exposures such as smoking, etc.). While a numbers of models are available with parametrizations for different OAR’s, it is as yet unclear which class of model (linear-non-threshold, plateau or bell-shaped) should be considered the most realistic. The full model by Schneider et al. [12] has the intuitive plausibility that high doses leading to cell kill would not be expected to be as cancerogenetic as lower doses resulting only in DNA damage without cell death. However, this hypothesis is not unanimously adopted, as some studies have also supported a linear relationship (compare Filippi et al. for a recent review [13]).

If the assumption of a bell-shaped model should hold true, however, it is highly relevant that all dose contributions should be summed together for secondary cancer estimation, rather than considering the effects individually. If the secondary cancer risk from imaging and from treatment were assessed separately from one another, the linear model would be applied to the small doses implicated from imaging. In principle, this approach would be invalid if the treatment dose is far from the linear dose-risk regime, so that the contributions from imaging and treatment are not additive in the simple sense. However, in some cases there is no way of performing a combined imaging plus treatment dose assessment for secondary cancer risk, e.g. if no information on the treatment dose exists and just general conclusions about different imaging systems and their comparison with each other are drawn [14].

While emphasizing that this approach should be avoided because of the presumed non-linear shape of the dose-risk curve, we perform this estimation to demonstrate in how far the results would be influenced by following this approach, as it is sometimes adopted in the literature. Table 6 gives the average dose to the lung and breast from imaging only for the different scenarios, and the resulting secondary cancer risk from the linear model (RED(D) = D). The estimated risk from this method is considerably higher (by a factor 3–4) than the risk resulting from the clinically realistic scenario in which imaging dose and treatment dose act together simultaneously. The possible non-linearity in the risk curve thus plays a significant role and different coinciding contributions to dose should be analysed together, if this is possible.

**Table 6:** Additional EAR from imaging only if imaging was considered separately from the treatment plan

	Scenario 2 (real)	Scenario 3 (200° kV-CBCT)	Scenario 4 (360° kV-CBCT)	Scenario 5 (6 MV axes)	Scenario 6 (6MV CBCT)
Left lung	0.26 ± 0.34 (0–5.25)	0.08 ± 0.59 (0–5.25)	0.29 ± 0.21 (0.06–5.25)	0.48 ± 0.31 (0.04–5.25)	2.48 ± 1.62 (0.26–5.25)
Right lung	0.25 ± 0.34 (0–1.07)	0.07 ± 0.48 (0–0.15)	0.18 ± 0.17 (0–0.48)	0.34 ± 0.21 (0.04–0.63)	2.28 ± 1.38 (0.24–4.32)
Left breast	0.89 ± 0.57 (0.31–1.41)	0.24 ± 0.10 (0.16–0.39)	1.74 ± 0.83 (1.05–2.96)	2.51 ± 1.27 (1.45–4.35)	10.27 ± 5.08 (6.37–17.7)
Right breast	0.78 ± 0.61 (0.23–1.52)	0.23 ± 0.11 (0.08–0.33)	1.71 ± 0.65 (0.97–2.33)	1.80 ± 0.70 (1.05–2.41)	10.60 ± 3.68 (6.37–13.98)

This table assumes that imaging was considered separately from the treatment dose. In this case, a linear risk model would be applied as imaging dose is in the low dose regime. Data are given as cases per 10^4^ PY, average ± standard deviation, range in braces

### Comparison with previous studies

A number of studies have estimated imaging dose for MV and kV modalities in either planar or CBCT geometries, although relatively few have considered pediatric patients. For the Varian On-Board Imager (OBI) system (Varian Medical Systems, Palo Alto, California, USA), Ding et al. [15] obtained doses to soft tissue of the order of 4–10 cGy (half-fan) and 3–9 cGy (full-fan mode) for pediatric patients with imaging in the head-and-neck region using the Vanderbilt-Monte-Carlo-Beam-Calibration algorithm. Similar dose values (1.9–10.5 cGy depending on the organ) were reported by Deng et al. [16] for 125 kVp voltage based on Monte Carlo methods. Measurements in an anthropomorphic phantom (adult and pediatric) [17] confirm this order of magnitude. Our calculated average organ doses for the kV imaging scenarios are larger than the original treatment plan dose by up to 20 cGy for daily imaging (up to 16 fractions, which means a few cGy per image). This is in good agreement with previous studies, which can be expected since dose calculations for our system also agreed well with those from Varian and Elekta (Elekta, Stockholm, Sweden) for other tumor indications.

Regarding secondary cancer risk estimates, the results from previous studies are rather varied depending on the assumed target localization, dose distribution and secondary cancer model (for reviews, see [18, 19] and references therein). Comparing our predictions with clinical data, Dörffel et al. [20] observed an absolute excess risk of developing secondary malignancies at the breast of 14.9 per 10,000 PY in a cohort study of 2548 patients treated for Hodgkin’s Lymphoma within 30 years’ follow-up, which agrees well with the range of values obtained in our study (2–26 cases per 10,000 PY). In their review, Kamran et al. [21] list an absolute risk for breast cancer after Hodgkin lymphoma of 37 per 10,000 PY; Schaapveld et al. [22] estimate an EAR of 20.5–29.3 per 10,000 PY for the lung and 44.7–65.0 per 10,000 PY for the breast. Since our prediction is for an attained age of 50 years and increases with higher age, the agreement is still plausible.

Only few studies have focused on secondary cancer risk from imaging in radiotherapy. Zhang et al. [23] presented imaging dose from Varian kV CBCT as a function of patient chest circumference, where organ mean dose per scan was of the order of 0.8–3 cGy (in agreement with our results), but used the BEIR VII model to transfer these values into relative risks for secondary cancer. As we have pointed out, the non-linear behaviour of the cancer risk curve should be accounted for by considering the combination of treatment and imaging dose where this is possible. For this high-dose regime, it is more common to use Schneider’s full mechanistic model; however, this is usually applied to treatment plans rather than IGRT scenarios [24]. To our knowledge, our study is the first one to include imaging scenarios in the radiotherapy treatment plan for an accurate assessment of additional cancer risk in the dose regime used for treatment.

### Limitations and implications of this study

In addition to the relatively small patient collective available for our study, it must be born in mind that the treatment plans were not particularly optimized to minimize secondary cancer risk, neither for optimal sparing of the female breast. Although these organs are taken into account as an organs at risk in our planning process, a compromise was made for best sparing of all healthy organs, so that the two sites at most risk for secondary cancer incidence were not prioritized over the other OAR’s. While all patients were treated with the involved field technique, the target volumes showed strong variation (PTV volumes 54–2419 cm^3^, average 1145 cm^3^). Most patients were treated using step-and-shoot IMRT (4–14 beams with between 30 and 70 segments), only very small target volumes (e.g., case 9, os ileum) were treated using 3D conformal radiotherapy (3D CRT with 2 beams). Volumetric modulated arc therapy (VMAT) plans were never used. The number of beams and segments used in the IMRT plans varied individually. In addition to setting objectives for the organs at risk, ring and normal tissue structures were created to form a dose gradient around the target volume.

Despite the large variability between the patients, the DVH metrics correspond to those reported by other authors. For example, Maraldo et al. [25] assessed normal tissue doses for different radiotherapy targets for patients with Hodgkin’s lymphoma. For the lungs, they found mean doses in the range of 30–52% of the prescribed dose (30 Gy in their study, i.e. 11.7–15.6 Gy) for mantle field and mediastinal irradiation and 2.2–4.0 Gy for neck and axilla irradiation, which agrees with the range of doses from our collective (1.6–18.7 Gy mean lung dose). Similarly, their values for the female breast were between 1.4 and 17 Gy depending on the treatment fields, corresponding to our dose range between 0.7 and 11.5 Gy.

Regarding margins for the PTV, in a previous unpublished study (similar to [8]) we analyzed positioning errors for a collective of children, resulting in a systematic population setup error of 0.7/1.0/− 0.2 mm in the anterior-posterior (AP)/left-right (LR) and superior−/inferior (SI) directions, a variation of population setup error of 1.7/2.6/2.5 mm (AP/LR/SI) and a population random error or 3.9/4.9/3.9 mm (AP/LR/SI), which yields a CTV-PTV margin of 8.2/11.4/10.2 mm according to the margin recipe by van Herk et al. [26]. In the clinical practice, between 10 and 15 mm margin are used at our department. In fact, the use of daily imaging would be expected to reduce the required margins, as the daily statistical error could be corrected before treatment and position close to the planned “ideal” position could be attained. In this case, a margin reduction would allow for better sparing of organs at risk and a possible decrease in secondary cancer risk. Therefore, the increase in dose from daily imaging might be counterbalanced by an sparing in dose due to improved positioning accuracy. Such a trade-off would be interesting to perform, but remains outside the scope of this study as a number of combinations (margin vs. imaging scenario) would need to be compared. Similar approaches have been presented for other treatment sites [11, 27]. As a rule-of-thumb, dose from kV imaging is relatively small and it might be expected that the positive influence of dose sparing from margin reduction would be dominant in this scenario. Regarding daily MV imaging, however, this might no longer be the case, and it is generally believed that a daily 6 MV CBCT scenario should be avoided unless in very limited indications (e.g., direct vicinity of critical OAR and high-risk target volume).

Finally, the present study focusses on a highly vulnerable collective (children with good long-term prognosis treated with relatively large fields and wide margins), so that the resulting estimated risk can be expected to be larger than for the most frequent radiotherapy treatments (adult patients with small fields), where improved positioning accuracy and small margins achieved by daily (kV) imaging might considerably reduce normal tissue complications without detrimentous effects on secondary cancer risk.

## Conclusions

If daily imaging is required, this can be performed with less than 1 case per 10^4^ PY additional cancer risk using kV CBCT or – if no other option exists – 6 MV as long as planar images are taken. Depending on the geometry of the X-ray tube (e.g., opposite to the treatment head), a further reduction on dose can be achieved for superficial organs such as the breasts by applying only a reduced-arc rotation (in our case, behind the patient’s back). When analyzing secondary cancer risk from imaging modalities, this should in the best case be combined with the treatment plan, as a separate analysis of only imaging dose in the linear range leads to an overestimation of the secondary cancer risk.

## Abbreviations

AP: Anterior-posterior
CBCT: Cone-beam CT
CRT: Conformal radiotherapy
CT: Computed tomography
DICOM: Digital Imaging and Communications in Medicine
DVH: Dose-volume histogram
EAR: Excess absolute risk
EPID: Electron portal imaging device
IBL: Imaging beam line
IGRT: Image-guided radiotherapy
LR: Left-right
MU: Monitor units
OAR: Organ at risk
OBI: On-Board Imager
PTV: Planning target volume
PY: Patient-years
RED: Risk equivalent dose
ROI: Region of interest
SI: Superior-inferior
SSD: Source-to-surface distance
TBL: Treatment beam line
TPS: Treatment planning system
VMAT: Volumetric modulated arc therapy
